# Protective effects of traditional Chinese medicine formula NaoShuanTong capsule on haemorheology and cerebral energy metabolism disorders in rats with blood stasis

**DOI:** 10.1080/13102818.2014.901678

**Published:** 2014-06-04

**Authors:** Hong Liu, Yao-yao Peng, Feng-yin Liang, Si Chen, Pei-bo Li, Wei Peng, Zhong-zheng Liu, Cheng-shi Xie, Chao-feng Long, Wei-wei Su

**Affiliations:** ^a^ Guangzhou Quality R & D Center of Traditional Chinese Medicine, Guangdong Key Laboratory of Plant Resources, School of Life Sciences, Sun Yat-sen University, Guangzhou, P.R. China; ^b^ Guangdong Zhongsheng Pharmaceutical Co. Ltd., Dongguan, P.R. China; ^c^ ^c^Guangzhou Blood Center, Guangzhou, P.R. China

**Keywords:** NaoShuanTong capsule, blood stasis, haemorheology, cerebral energy metabolism, Chinese medicine formula

## Abstract

NaoShuanTong capsule (NSTC), an oral traditional Chinese medicine formula, is composed of *Pollen Typhae*, *Radix Paeoniae Rubra*, *Rhizoma Gastrodiae*, *Radix Rhapontici* and *Radix Curcumae*. It has been widely used to treat ischemic stroke in clinic for many years in China. In addition to neuronal apoptosis, haemorheology and cerebral energy metabolism disorders also play an important role in the pathogenesis and development of ischemic stroke. The present study was designed to evaluate the *in vivo* protective effects of NSTC on haemorheology and cerebral energy metabolism disorders in rats with blood stasis. Sixty specific pathogen-free sprague-dawley rats, male only, were randomly divided into six groups (control group, model group, aspirin (100 mg/kg/d) group, NSTC low-dose (400 mg/kg/d) group, NSTC intermediate-dose (800 mg/kg/d) group, NSTC high-dose (1600 mg/kg/d) group) with 10 animals in each. The rats except those in the control group were placed in ice-cold water (0–4 °C) for 5 min during the time interval (4 h) of two adrenaline hydrochloride injections (0.8 mg/kg) to induce blood stasis. After treatment, whole blood viscosity at three shear rates, plasma viscosity and erythrocyte sedimentation rate significantly decreased in NSTC intermediate- and high-dose groups; erythrocyte aggregation index and red corpuscle electrophoresis index significantly decreased in all the three dose NSTC groups. Moreover, treatment with high-dose NSTC could significantly improve Na^+^–K^+^ adenosine triphosphatase (ATPase) and Ca^2+^ ATPase activity, as well as lower lactic acid level in brain tissues. These results demonstrated the protective effects of NSTC on haemorheology and cerebral energy metabolism disorders, which may provide scientific information for the further understanding of mechanism(s) of NSTC as a clinical treatment for ischemic stroke. Furthermore, the protective effects of activating blood circulation as observed in this study might create valuable insight for the utilisation of NSTC to be a feasible alternative therapeutic agent for patients with blood stasis.

## Introduction

In addition to neuronal apoptosis, haemorheology and cerebral energy metabolism disorders also play an important role in the pathogenesis and development of ischemic stroke (IS). Haemorheological disturbances, promoting thrombogenesis and atherosclerosis, might occur in more than 40% of patients with IS.[1] Elevated whole blood viscosity (WBV), plasma viscosity (PV) and increased red blood cell (RBC) aggregation have been proved to be important risk factors in cerebrovascular diseases.[2] Maintenance of blood flow rate is a critical factor for tissue oxygen and substrate supply.[3] The limited availability of glucose and oxygen directly impaired oxidative metabolism in severely ischemic regions of the affected tissue, as well as led to rapid changes in ATP and other energy-related metabolites.[4] Moreover, it was reported that Buyang Huanwu Decoction, a different Chinese medicine formula, could improve energy metabolism in mice with cerebro-ischemic injury by increasing the Na^+^–K^+^ ATPase activity.[5]

Blood stasis, a common syndrome in traditional Chinese medicine, an indicator of haemorheological abnormalities, is characterised by the decrease of blood flow velocity and the disturbance of the normal blood flow.[6] Once blood stasis developed, the blood circulation would further be affected and thus lead to new pathological changes.[7]

NaoShuanTong capsule (NSTC), an oral traditional Chinese medicine formula, is composed of *Pollen Typhae*, *Radix Paeoniae Rubra*, *Rhizoma Gastrodiae*, *Radix Rhapontici* and *Radix Curcumae* in a ratio of 3:2:2:1:1. It has been widely used in clinic to treat IS by activating blood circulation and improvement of neurological functional recovery for many years in China. However, to the best of our knowledge, no studies concerning the effects of NSTC on haemorheology and cerebral energy metabolism disorders have been reported so far. The present study was designed to evaluate the *in vivo* protective effects of NSTC on haemorheology and cerebral energy metabolism disorders in rats with blood stasis.

## Materials and methods

### Materials and reagents

NSTC sample for the experiment (Batch No: 20110406) was provided by Zhongsheng Pharmaceutical Co. Ltd. (Guangdong, China). The preparation process of NSTC sample was as follows: soaking *Radix* *Curcumae* in five times the volume of ethanol (80%, v/v) for 12 h, reflux extracting two times, each time for 1 h, mixing extractions, filtering and gathering residue individually; refluxing *Radix Paeoniae Rubra* two times with five times the volume of ethanol (70%, v/v), each time for 1 h, mixing extractions, filtering, recovering ethanol, concentrating to obtain soft extract; mixing *Pollen Typhae* (loaded in cloth bag), *Rhizoma Gastrodiae*, *Radix Rhapontici* and *Radix Curcumae* residue, soaking in 10 times the volume of warm water (80 °C) for 2 h, decocting two times, each time for 1 h, mixing extractions, filtering, mixing with *Radix Curcumae* extract, recovering ethanol, concentrating to soft extract; mixing with *Radix Paeoniae Rubra* soft extract to obtain the NSTC sample. Then NSTC sample was dissolved in normal saline into 40, 80 and 160 mg/ml for treatment. Adrenalin hydrochloride (Adr) was purchased from Shanghai Harvest Pharmaceutical Co. Ltd. (State medical permission No: H31021062, Batch No: 20111109). Aspirin (ASP) was purchased from JilinLuwang pharmaceutical Co. Ltd. (State medical permission No: H22025784, Batch No: BTA7WH2). Na^+^–K^+^ ATPase assay kit, Ca^2+^ ATPase assay kit, lactic acid (LAC) assay kit and bicinchonininc acid (BCA) Protein assay kit were purchased from Nanjing Jiancheng Bioengineering Institute (Nanjing, China). Solvents used for high performance liquid chromatography (HPLC) were of HPLC grade. All other agents used were of the commercially available grade.

### High performance liquid chromatography analysis of NSTC

**Figure 1.**
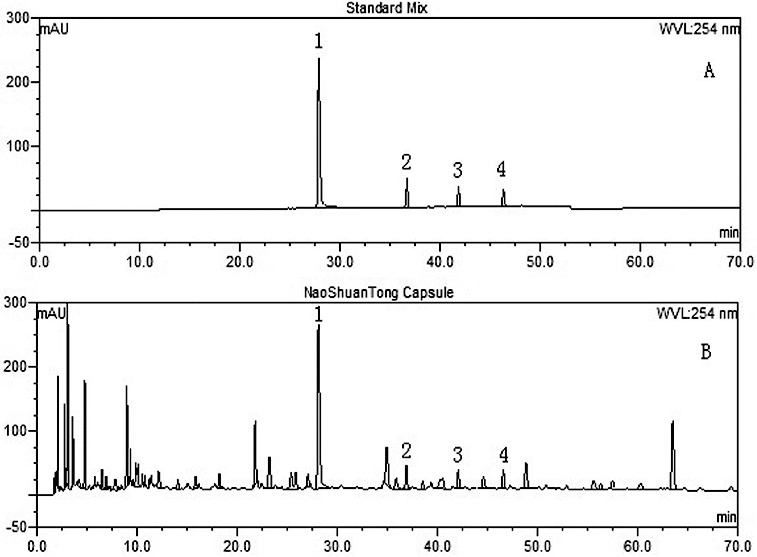
High performance liquid chromatography (HPLC) of standard mix (**A**) and NaoShuanTong capsule (B) using ultraviolet absorbance detection at wavelength 254 nm. 1: Paeoniflorin; 2: Ecdysterone; 3: Typhaneoside; 4: Isorhamnetin-3-O-neohesperidoside.

The major components of NSTC are flavonoids, glycosides, phenols, ketosteroids and so on.[8–10] Prior to treating the experimental rats, NSTC sample used in this study was analysed to ensure the quality control, with an Ultimate 3000 HPLC system (Dionex, S/N: 8043731 USA) consisting of DGP-3600SD pump, SRD-3600 degasser, WPS-3000SL autosampler, TCC-3000RS column compartment and DAD-3000 diode array detector. The original data were calculated and processed with Chromeleon 6.8 Software. An Ultimate AQ-C_18_ (150 × 4.6 mm, 3 μm) column was used for the analysis, and acetonitrile (A)–tetrahydrofuran (B)–0.05% phosphoric acid (C) were served as the mobile phase with linear gradient elution in 70 min (A: 2%→20%, B: 0%→10%, C: 98%→70%). The flow rate was 1.1 ml/min with ultraviolet absorbance detection at 254 nm. The operation was carried out at 20 °C. Paeoniflorin, ecdysterone, typhaneoside and isorhamnetin-3-O-neohesperidoside were identified and quantified by the comparison of retention time and ultraviolet spectra with those of standard mix, as shown in Figure 1.

### Animal treatment

Sixty specific pathogen free (SPF) Sprague-dawley rats, male only, weighing 180–220 g were obtained from Guangdong medical experimental animal centre (certification no: SCXK-(Yue) 2008-0002) and raised in SPF laboratory of ocean and traditional Chinese medicine laboratory of Sun Yat-sen University (License: SCXK-(Yue) 2009-0020). Rats were kept under a 12 h light–dark cycle with the temperature of 22 ± 2 °C and relative humidity of 50%–65%, and had free access to standard laboratory pellet food and water. Animal welfare and experimental procedures were carried out in accordance with the guide for the care and use of laboratory animals (National Research Council of USA, 1996) and approved by Animal Care and Use Committee of School of Life Sciences, Sun Yat-sen University (Permission No: 2012052501).

### Experimental model

Sixty rats were randomly divided into six groups with 10 animals in each. Group 1 was the control: rats were given blank solvent (normal saline) as the vehicle at the same volume. Group 2 was the model: rats were given blank solvent (normal saline) at the same volume. Group 3 was model + ASP: rats were given ASP 100 mg/kg/d. Group 4 was model + low-dose NSTC: rats were given NSTC 400 mg/kg/d, which is regarded as the clinically equivalent dosage for adults. Group 5 was model + intermediate-dose NSTC: rats were given NSTC 800 mg/kg/d. Group 6 was model + high-dose NSTC: rats were given NSTC 1600 mg/kg/d. All treatments were performed by gavage with a volume of 10 ml/kg/d and were administered 10 times with an interval of 24 h.

After the 10th administration, rats except those in the control group were subcutaneously injected with Adr (0.8 mg/kg). After 2 h, rats except those in control group were kept in ice-cold water (0–4 °C) for 5 min and 2 h later were re-injected with Adr (0.8 mg/kg) subcutaneously to induce blood stasis. Rats in the control group received two normal saline injections subcutaneously at the same volume.[11,12] Then, all the rats were fasted for 12 h before the last administration.

### Blood collection

Rats were anesthetised with chloral hydrate (300 mg/kg) 1 h after the last administration. Blood was drawn from the abdominal aortas and collected into plastic tubes with 3.8% sodium citrate (citrate/blood: 1/9, v/v) for the determination of haemorheological parameters including WBV, erythrocyte sedimentation rate (ESR), erythrocyte aggregation index (EAI) and red corpuscle electrophoresis index (RCEI). Plasma was separated from blood sample at 3000 rpm for 10 min to detect PV. All the procedures including blood collection and parameters determination were completed within 3 h after blood collection.

### Viscosity and ESR determination

A total of 800 μl blood sample was used to detect EAI, RCEI and WBV (5, 50 and 200 s^−1^ shear rate), and plasma separated from 1500 μl blood sample was used to measure PV (120 s^−1^ shear rate). Determination of the above parameters was conducted with a cone-plate viscometer (LBY-N6B, Precil. Co., China) maintained at 37 °C. A total of 1000 μl blood sample was put into upright westergren tube. The rate of RBC falling to the bottom of the tube was observed and reported with an ESR Automatic Monitor (LBY-XC40 ESR Automatic Monitor Precil. Co., China).

### Determination of Na^+^–K^+^ ATPase, Ca^2+^ ATPase activity and LAC content in brain tissues

The animals were sacrificed and brain tissues were collected for the determination of the Na^+^–K^+^ ATPase, Ca^2+^ ATPase activity and LAC content. The brain tissue was homogenised with normal saline (brain tissue/normal saline: 1/9, v/v) and then centrifuged at 1000× *g* for 10 min. A 0.2 ml supernatant was mixed with 0.8 ml normal saline to form a new mixed solution that was used for the determination of protein content with bicinchonininc acid (BCA) method. The LAC content was measured with an LAC assay kit according to the manufacturer's instructions. The Na^+^–K^+^ ATPase activity and Ca^2+^ ATPase activity were evaluated by measuring inorganic phosphate (Pi) generation from ATP in brain homogenates at 37 °C.[13]

### Statistical analysis

The data were expressed as mean ± standard deviation (SD). One-way analysis of variance, Student's *t*-test and Dunnett's multiple comparisons test were used under SPSS 18.0 for comparison of the results among the groups. *P*-value less than 0.05 or 0.01 represented statistical significance.

## Results and discussion

Haemorheology, often observed in patients with IS, is commonly used to evaluate the clinical therapeutic effects of the relevant drugs.[14,15] Haemorheological abnormality was reported to play an important role in the pathogenesis and development of cerebrovascular diseases.[16] Due to the important role of haemorheological parameters in the regulation of cerebral blood flow, PV, WBV and enhanced erythrocyte aggregation were proved to correlate with decreased cerebral blood flow.[1] WBV, the reflection of intrinsic resistance of blood flowing in vessels, has been documented to rise in IS patients by numerous studies.[17–19] As shown in Table 1, WBV at three shear rates significantly decreased in intermediate- and high-dose NSTC groups (*P* < 0.05, *P* < 0.01). In Table 2, the significant decrease of both EAI and RCEI, in line with the change of WBV, could be observed after treatment with the entire three doses of NSTC (*P* < 0.01). It is understood that WBV at low shear rates, EAI and RCEI all can reflect the degree of aggregation among RBCs. Therefore, these results suggested that the improvement of blood circulation by NSTC might be associated with the reduction of RBC aggregation.

**Table 1.** Effects of NSTC on WBV and PV.

		WBV (mPa·s)			
Group	Dose (mg/kg/d)	5 s^−1^	50 s^−1^	200 s^−1^	PV 120 s^−1^ (mPa·s)
Control	NS	8.95 ± 2.17	6.01 ± 0.92	4.68 ± 0.46	1.04 ± 0.05
Model	NS	14.19 ± 2.01^##^	7.36 ± 0.86^##^	5.67 ± 0.54^##^	1.23 ± 0.01^##^
Asp	100	11.08 ± 3.57*	6.51 ± 1.18	5.31 ± 0.81	1.18 ± 0.03**
NSTC	400	11.25 ± 2.16*	6.74 ± 0.91	5.39 ± 0.33	1.21 ± 0.02*
NSTC	800	11.09 ± 2.67*	6.37 ± 0.73*	5.16 ± 0.37*	1.20 ± 0.03*
NSTC	1600	10.05 ± 2.62**	6.19 ± 0.54**	5.12 ± 0.42*	1.18 ± 0.05*

Note: All the data were shown as the mean ± SD, *n* = 10.

NSTC: NaoShuanTong Capsule; ASP: aspirin; WBV: whole blood viscosity; PV: plasma viscosity.

Control group and model group received the same volume of normal saline (NS) for the treatment (10 ml/kg/d). ^#^ *P* < 0.05 and ^##^ *P* < 0.01 when compared with control group. *P < 0.05 and **P < 0.01 when compared with model group.

**Table 2.** Effects of NSTC on ESR, EAI and RCEI.

Group	Dose (mg/kg/d)	ESR (mm/h)	EAI	RCEI
Control	NS	0.85 ± 0.39	1.94 ± 0.36	4.09 ± 0.67
Model	NS	4.24 ± 1.40^##^	2.54 ± 0.25^##^	5.39 ± 0.36^##^
ASP	100	2.06 ± 1.37**	2.09 ± 0.51**	4.55 ± 0.98*
NSTC	400	3.59 ± 1.76	2.14 ± 0.33**	4.52 ± 0.67**
NSTC	800	2.62 ± 0.89**	2.02 ± 0.34**	4.36 ± 0.82**
NSTC	1600	1.94 ± 0.89**	1.96 ± 0.46**	4.12 ± 0.96**

Note: All the data were shown as the mean ± SD, *n* = 10.

NSTC: NaoShuanTong Capsule; ASP: aspirin; ESR: erythrocyte sedimentation rate; EAI: erythrocyte aggregation index; RCEI: red corpuscle electrophoresis index.

Control group and model group received the same volume of normal saline (NS) for the treatment (10 ml/kg/d). ^#^ *P* < 0.05 and ^##^ *P* < 0.01 when compared with control group. *P < 0.05 and **P < 0.01 when compared with model group.

The RBCs account for almost 50% of blood volume and constitute the majority of the cellular content in blood. ESR, the sedimentation rate of RBCs, is the reflection of RBC aggregation and plays an important role in homeostasis due to the local interaction between platelets and the endothelial wall.[20] It has been reported that ESR was elevated by 68% in 241 consecutive patients with acute IS [21] and considered as the effective factors in IS prognosis.[22] In the present study, treatment with NSTC showed a significantly lower ESR compared with the model group (Table 2), which demonstrated the effective performance of NSTC to decrease the RBC aggregation. The apparent viscosity of the whole blood is also determined partly by the PV level. The significant decrease of PV observed in NSTC low-, intermediate- and high-dose groups (Table 1) demonstrated that NSTC had the potential to prevent the increase of PV level in rats with blood stasis. The results suggested that the decrease of apparent blood viscosity after NSTC treatment might also be mediated via the inhibition of the increase in PV level. Therefore, data from the present study revealed that the suppression of RBC aggregation and PV level might be two of the potential mechanisms responsible for NSTC alleviation of haemorheological disorders.

**Figure 2.**
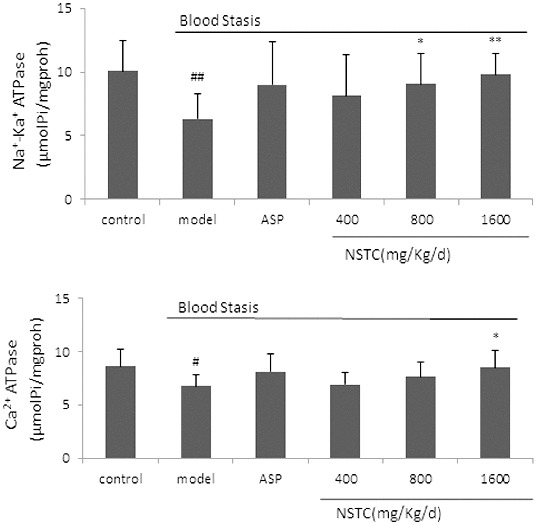
Effect of NSTC on the Na^+^–K^+^ ATPase and Ca^2+^ ATPase activity in brain tissues. NSTC: NaoShuanTong capsule; ASP: aspirin. Groups: control group, model group, ASP group (100 mg/kg/d) and three NSTC groups (400, 800 and 1600 mg/kg/d). Control group and model group received the same volume of normal saline (NS) for the treatment (10 ml/kg/d). Each bar represents the Na^+^–K^+^ ATPase and Ca^2+^ ATPase activity as mean ± SD, *n* = 10. Note: ^#^ *P* < 0.05 and ^##^ *P* < 0.01 when compared with control group. *P < 0.05 and **P < 0.01 when compared with model group.

In addition to haemorheological disorders, cerebral energy metabolism also plays pivotal roles in IS. Decreased blood flow reduces the availability of oxygen, provoking massive glycolysis.[3] Numerous studies suggested that hypoxia–ischemia could affect a wide range of cellular transport mechanisms like Na^+^–K^+^ ATPase activity.[23–26] ATP is a molecule universally used to energise cellular activities, particularly in brain tissues, which accounted for 20% of the systemic energy consumption.[27] Marked changes in ATP and related energy metabolites develop quickly in response to the limitations of oxygen and glucose.[28] Na^+^–K^+^ ATPase and Ca^2+^ ATPase are components of the plasma membrane and transport ions using ATP hydrolysis. The level of ATP in brain tissues can be observed through the Na^+^–K^+^ ATPase and Ca^2+^ ATPase activity. It has been reported that Na^+^–K^+^ ATPase consumes 40%–50% of the ATP generated in the brain [29] and the decrease in this enzyme activity is considered to reflect the consequences of the neurocyte energetic metabolism alterations caused by cerebral ischemia.[30] Evidence suggested that Na^+^–K^+^ ATPase activity, which was very sensitive to hypoxia would be reduced or insufficient to maintain ionic balances during and immediately after episodes of ischemia and Na^+^–K^+^ ATPase inhibition persisted during reoxygenation.[24,31,32] The excessive retention of sodium results in osmotic swelling and possible cellular lysis and causes a failure of sodium–calcium exchange system that is responsible for a build-up of intracellular calcium.[32] After administration, NSTC significantly increased Na^+^–K^+^ ATPase and Ca^2+^ ATPase activity in brain tissues as shown in Figure 2. Therefore, the improvement of NSTC on cerebral energy metabolism might be in consequence of the enhancement of Na^+^–K^+^ ATPase and Ca^2+^ ATPase activity in brain tissues.

**Figure 3.**
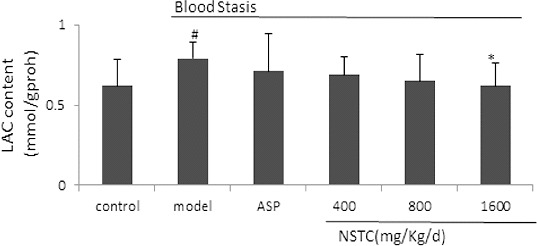
Effect of NSTC on the LAC content in brain tissues. NSTC: NaoShuanTong Capsule; ASP: aspirin; LAC: lactic acid. Groups: control group, model group, ASP group (100 mg/kg/d) and three NSTC groups (400, 800 and 1600 mg/kg/d). Each bar represents the LAC content as mean ± SD, *n* = 10. Note: ^#^ *P* < 0.05 and ^##^ *P* < 0.01 when compared with control group. *P < 0.05 and **P < 0.01 when compared with model group.

LAC has been commonly used as nonspecific but sensitive marker of acute cerebrovascular diseases response. In severe ischemia and tissue hypoxia, anaerobic glycolysis leads to LAC accumulation. Twenty-minute severe forebrain ischemia was associated with a marked increase in LAC, and excessive level of lactic acidosis during ischemia or hypoxia would severely hamper metabolic and functional restitution upon subsequent recirculation and reoxygenation, leading to the irreversible damage of cell morphology.[33,34] Therefore, there will be less damage if severe tissue lactic acidosis is hindered. As shown in Figure 3, the LAC content decreased significantly after NSTC treatment, suggesting that NSTC could regulate LAC content in brain tissues of rats, which might be responsible for its protective effects against IS.

## Conclusions

The results demonstrated that NSTC could improve blood circulation to remove blood stasis, which might be associated with the inhibition of aggregation among RBCs and PV. NSTC regulation of cerebral energy metabolism disorders could also be observed including the increase of Na^+^–K^+^ ATPase, Ca^2+^ ATPase activity and decrease of LAC content. Overall, both haemorheology and cerebral energy metabolism disorders contribute to the pathogenesis and development of IS. As evidenced in this study, the protective effects on haemorheology and cerebral energy metabolism disorders might provide scientific information for the further understanding of mechanism(s) of NSTC as a clinical treatment for IS. Furthermore, the ability to activate blood circulation might create valuable insight for utilising NSTC as a feasible alternative therapeutic agent for patients with blood stasis.

## Note

1. Contributed equally to this work.
